# Poly[tris­(μ-4,4′-bi­pyridine-κ^2^ *N*:*N*′)bis(dimethyl sulfoxide-κ*O*)tetra­kis­(thiocyanato-κ*N*)dicobalt(II)]

**DOI:** 10.1107/S1600536814013555

**Published:** 2014-06-18

**Authors:** Surasak Kaenket, Pongthipun Phuengphai, Chaveng Pakawatchai, Sujittra Youngme

**Affiliations:** a Materials Chemistry Research Unit, Department of Chemistry and Center of Excellence for Innovation in Chemistry, Faculty of Science, Khon Kaen University, Khon Kaen 40002, Thailand; b Department of Fundamental Science, Faculty of Science and Technology, Surindra Rajabhat University, Surin 32000, Thailand; c Department of Chemistry, Faculty of Science, Prince of Songkla University, Hat Yai, Songkhla 90112, Thailand

## Abstract

The asymmetric unit of the title compound, [Co_2_(NCS)_4_(C_10_H_8_N_2_)_3_(C_2_H_6_OS)_2_]_*n*_, consists of one Co^II^ atom, two thio­cyanate anions, one dimethyl sulfoxide mol­ecule and one and a half 4,4′-bi­pyridine mol­ecules. The half-molecule is completed by inversion symmetry. The Co^II^ atom is coordin­ated in a distorted octa­hedral geometry by two N atoms from two thio­cyanate anions, one O atom from dimethyl sulfoxide as a terminal ligand and three N atoms from three 4,4′-bi­pyridine mol­ecules as bridging ligands linking the cations, with a Co⋯Co separation of 11.5964 (5) Å. This generates a two-dimensional structure parallel to (-103). A C—H⋯S hydrogen bond links the layers into a three-dimensional supra­molecular framework. The layers are stacked in an *ABC* fashion preventing the occurrence of inter­layer void space and hence leading to the absence of lattice solvent and/or organic guest mol­ecules in the structure.

## Related literature   

For related coordination polymers with ligands such as pyrazine, pyrimidine, 4,4′-bi­pyridine and SCN^−^, see: Wriedt & Näther (2009 ▶, 2010 ▶); Wriedt *et al.* (2009 ▶); Yao & Wang (2009 ▶).
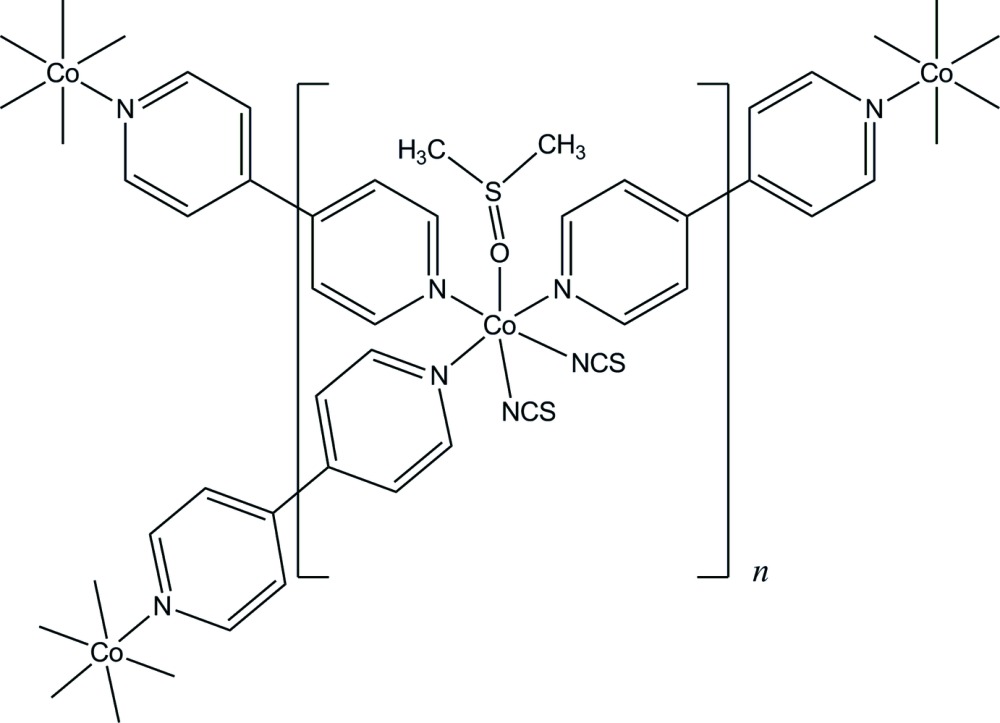


## Experimental   

### 

#### Crystal data  

[Co_2_(NCS)_4_(C_10_H_8_N_2_)_3_(C_2_H_6_OS)_2_]
*M* *_r_* = 974.98
Monoclinic, 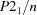

*a* = 11.0772 (3) Å
*b* = 16.9999 (2) Å
*c* = 11.6843 (3) Å
β = 103.628 (1)°
*V* = 2138.34 (8) Å^3^
*Z* = 2
Mo *K*α radiation
μ = 1.12 mm^−1^
*T* = 273 K
0.40 × 0.16 × 0.10 mm

#### Data collection  

Bruker SMART APEX CCD diffractometer
Absorption correction: multi-scan (*SADABS*; Bruker, 2000 ▶) *T*_min_ = 0.591, *T*_max_ = 0.894
14262 measured reflections
5584 independent reflections
3936 reflections with *I* > 2σ(*I*)
*R*_int_ = 0.032

#### Refinement  

*R*[*F* ^2^ > 2σ(*F* ^2^)] = 0.043
*wR*(*F* ^2^) = 0.109
*S* = 1.01
5584 reflections
264 parameters
H-atom parameters constrained
Δρ_max_ = 1.02 e Å^−3^
Δρ_min_ = −0.58 e Å^−3^

### 

Data collection: *SMART* (Bruker, 2000 ▶); cell refinement: *SAINT* (Bruker, 2000 ▶); data reduction: *SAINT*; program(s) used to solve structure: *SHELXS97* (Sheldrick, 2008 ▶); program(s) used to refine structure: *SHELXL97* (Sheldrick, 2008 ▶); molecular graphics: *Mercury* (Macrae *et al.*, 2008 ▶); software used to prepare material for publication: *publCIF* (Westrip, 2010 ▶).

## Supplementary Material

Crystal structure: contains datablock(s) I. DOI: 10.1107/S1600536814013555/is5365sup1.cif

Structure factors: contains datablock(s) I. DOI: 10.1107/S1600536814013555/is5365Isup2.hkl

CCDC reference: 1007744

Additional supporting information:  crystallographic information; 3D view; checkCIF report

## Figures and Tables

**Table 1:** Selected bond lengths (Å)

Co1—N7	2.080 (2)
Co1—N6	2.102 (2)
Co1—O1	2.1234 (19)
Co1—N3	2.2187 (19)
Co1—N2	2.244 (2)
Co1—N1	2.2551 (19)

**Table 2:** Hydrogen-bond geometry (Å, °)

*D*—H⋯*A*	*D*—H	H⋯*A*	*D*⋯*A*	*D*—H⋯*A*
C1—H1⋯S1^i^	0.93	2.82	3.596 (3)	141

Symmetry code: (i) 
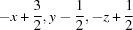
.

**Fig. 1.**
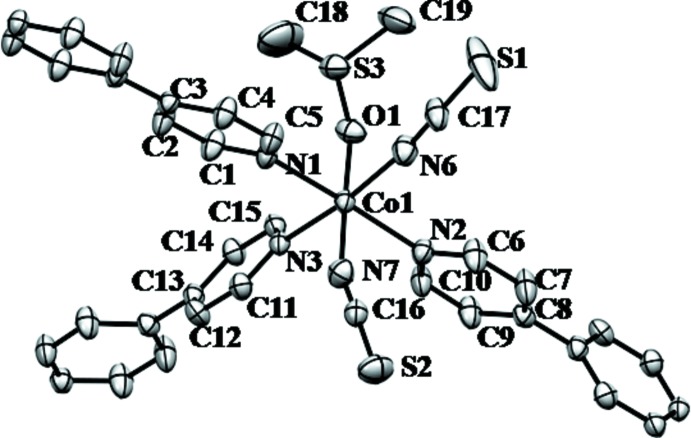
A view of the local coordination of the CoII in the title compound, showing the atom-numbering scheme. Displacement ellipsoids are drawn at the 50% probability level. Hydrogen atoms are omitted for clarity.

**Fig. 2.**
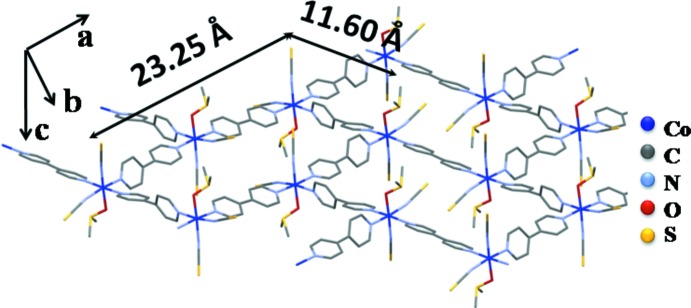
A partial packing diagram of the title compound, showing the two-dimensional structure via 4,4'-bpy bridges generating rectangular spaces.

**Fig. 3.**
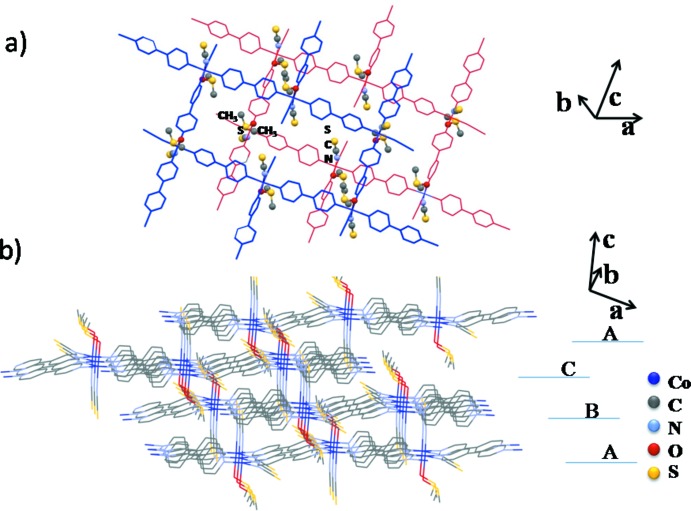
A partial packing diagram of the title compound, showing that terminal ligands, DMSO and SCN- fill up the rectangular space of adjacent layer (a) and ABC arrangement structure layers prevent the occurrence of the channel along C axis (b).

**Fig. 4.**
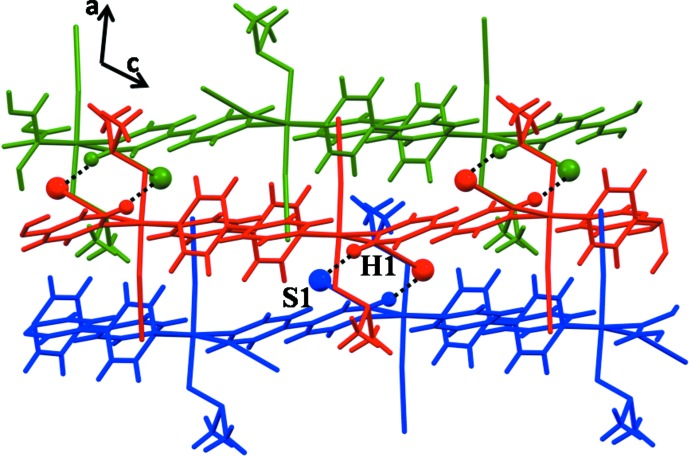
The extended structure of the title compound illustrates the hydrogen bonds (dotted lines) between H1 and S1 (-x + 3/2, y - 1/2, -z + 1/2) linking two-dimensional layers leading to a three-dimensional supramolecular framework. The adjacent layers are shown in different colours.

## supplementary crystallographic information

### S1. Comment

Metal organic frameworks can be prepared in variety methods and there are many effects influencing their structures. The solvent used in preparing is one of the most important effects on the structures. The influence of the solvent on the structure has been widely studied, for example in the study of iron(II) thiocyanato coordination polymers based on 4,4'-bipyridine using methanol as a solvent (Wriedt & Näther, 2010). This finding suggested that if more solvent and higher concentration of N-donor ligand were applied the structure is likely to involve with solvent coordination and the different metal to organic ligand ratio. The solvent has the influence on both metal to organic ligand ratio and the arrangement of the organic linker leading to the variation of the dimension and topology of the network (Yao & Wang, 2009). In addition, the type of N-donor organic linkers also affect the structure (Wriedt & Näther, 2009; Wriedt *et al.*, 2009).

Of interest to us was this effect. A new structure with the different metal to N-donor organic ligand ratio might also be possible by alteration of the solvent, type of N-donor organic ligand, and the metal to N-donor ligand ratio in the preparation. In this contribution, we present synthesis and structural characterization of a two-dimensional framework of poly[µ-tris(4,4'-bipyridine)di(dimethyl sulfoxide)tetrathiocyanato-*N*-dicobalt(II)] (I)

The asymmetric unit of the title compound consists of one Co^II^ centre, two SCN^-^ anions, one and a half 4,4'-bpy molecules and one DMSO molecule (Fig. 1). The Co^II^ is surrounded by two N atoms from terminal SCN^-^ groups, one O atom from DMSO and three N atoms from three 4,4'-bpy (Table 1). The 4,4'-bpy acts as a bridge linking metal centres and generates a two-dimensional structure with rectangular spaces (11.60 x 23.25 Å) within layer (Fig. 2). Due to the arrangement of the linker and metal to N-donor organic ligand ratio of 1:1.5, the space within the layer is twice as compared to the related two-dimensional compound {[Fe(4,4'-bpy)_2_(SCN)_2_](MeOH)_2_}_n_ (Wriedt & Näther, 2010). The layers are stacked in an ABC fashion (Fig. 3). The plane parallel to the layer is (103). The metal atoms in one layer sit above or below the rectangular spaces. As a result, the terminal SCN^-^ and DMSO ligands arrange approximately perpendicular to the layer plane and fill up the spaces between adjacent layers. This arrangement of the layers is in the ABC fashion preventing the occurrence of the interlayer spaces along the crystallographic c axis and hence leading to the absence of lattice solvent and/or organic guest molecules in the interlayer spaces (Fig. 3). In addition, the extended structure of I has been illustrated (Fig. 4). The hydrogen bonds between H1 and S1 link the layers with the distance of 2.82 Å (Table 2). As a result, these layers are assembled into a three-dimensional supramolecular framework.

### S2. Experimental

Compound I was synthesized by direct method in a molar ratio of 1:3:1 of Co(NO_3_)_2_·6H_2_O, 4,4'-bpy and KSCN, respectively. To prepare the reaction mixture, Co(NO_3_)_2_·6H_2_O (0.5 mmol, 0.15 g) and KSCN (0.5 mmol, 0.05 g) were dissolved in water (10 mL). Then 10 ml of ethanoic solution of 4,4'-bpy (1.5 mmol, 0.23 g) was added. The mixture was stirred, then 10 mL of DMSO and 0.5 mL of 6 M HNO_3_ was slowly added to assist dissolution.The mixture was then heated at 60 °C for 15 mins. It was set at room temperature for a slow evaporation. After 15 days, pink crystals were obtained.

### S3. Refinement

C-bound H atoms were positioned geometrically, with C—H = 0.93 (aromatic) or 0.96 Å (methyl), and included as riding atoms, with *U*_iso_(H) = 1.5*U*_eq_ (C) for methyl groups and 1.2*U*_eq_(C) otherwise.

### Crystal data

**Table:**

[Co_2_(NCS)_4_(C_10_H_8_N_2_)_3_(C_2_H_6_OS)_2_]	*Z* = 2
*M**_r_* = 974.98	*F*(000) = 1000
Monoclinic, *P*2_1_/*n*	*D*_x_ = 1.514 Mg m^−^^3^
Hall symbol: -P 2yn	Mo *K*α radiation, λ = 0.71073 Å
*a* = 11.0772 (3) Å	µ = 1.12 mm^−^^1^
*b* = 16.9999 (2) Å	*T* = 273 K
*c* = 11.6843 (3) Å	Block, pink
β = 103.628 (1)°	0.40 × 0.16 × 0.10 mm
*V* = 2138.34 (8) Å^3^	

### Data collection

**Table:**

Bruker SMART APEX CCD diffractometer	5584 independent reflections
Radiation source: fine-focus sealed tube	3936 reflections with *I* > 2σ(*I*)
Graphite monochromator	*R*_int_ = 0.032
phi and ω scans	θ_max_ = 29.8°, θ_min_ = 2.2°
Absorption correction: multi-scan (*SADABS*; Bruker, 2000)	*h* = −14→14
*T*_min_ = 0.591, *T*_max_ = 0.894	*k* = −16→22
14262 measured reflections	*l* = −12→15

### Refinement

**Table:**

Refinement on *F*^2^	Primary atom site location: structure-invariant direct methods
Least-squares matrix: full	Secondary atom site location: difference Fourier map
*R*[*F*^2^ > 2σ(*F*^2^)] = 0.043	Hydrogen site location: inferred from neighbouring sites
*wR*(*F*^2^) = 0.109	H-atom parameters constrained
*S* = 1.01	*w* = 1/[σ^2^(*F*_o_^2^) + (0.0522*P*)^2^ + 1.1491*P*] where *P* = (*F*_o_^2^ + 2*F*_c_^2^)/3
5584 reflections	(Δ/σ)_max_ < 0.001
264 parameters	Δρ_max_ = 1.02 e Å^−^^3^
0 restraints	Δρ_min_ = −0.58 e Å^−^^3^

### Special details

**Table:**

Geometry. All e.s.d.'s (except the e.s.d. in the dihedral angle between two l.s. planes) are estimated using the full covariance matrix. The cell e.s.d.'s are taken into account individually in the estimation of e.s.d.'s in distances, angles and torsion angles; correlations between e.s.d.'s in cell parameters are only used when they are defined by crystal symmetry. An approximate (isotropic) treatment of cell e.s.d.'s is used for estimating e.s.d.'s involving l.s. planes.
Refinement. Refinement of *F*^2^ against ALL reflections. The weighted *R*-factor *wR* and goodness of fit *S* are based on *F*^2^, conventional *R*-factors *R* are based on *F*, with *F* set to zero for negative *F*^2^. The threshold expression of *F*^2^ > σ(*F*^2^) is used only for calculating *R*-factors(gt) *etc*. and is not relevant to the choice of reflections for refinement. *R*-factors based on *F*^2^ are statistically about twice as large as those based on *F*, and *R*- factors based on ALL data will be even larger.

### Fractional atomic coordinates and isotropic or equivalent isotropic displacement parameters (Å^2^)

**Table:**

	*x*	*y*	*z*	*U*_iso_*/*U*_eq_	
Co1	0.61610 (3)	0.238576 (18)	0.34507 (3)	0.02619 (10)	
S3	0.74846 (7)	0.21001 (5)	0.14197 (7)	0.04276 (18)	
S2	0.57089 (8)	0.31500 (5)	0.72918 (8)	0.0535 (2)	
S1	0.93773 (9)	0.42788 (7)	0.32672 (16)	0.1102 (6)	
N3	0.47394 (17)	0.14430 (11)	0.30393 (18)	0.0268 (4)	
N2	0.47009 (19)	0.33263 (12)	0.30583 (19)	0.0309 (5)	
N1	0.76429 (18)	0.14581 (12)	0.39797 (18)	0.0303 (4)	
O1	0.62030 (17)	0.22923 (12)	0.16482 (17)	0.0396 (4)	
N7	0.6038 (2)	0.25004 (14)	0.5192 (2)	0.0394 (5)	
C15	0.4133 (2)	0.12562 (14)	0.1932 (2)	0.0297 (5)	
H15	0.4315	0.1541	0.1314	0.036*	
N6	0.7569 (2)	0.32360 (14)	0.3613 (2)	0.0435 (6)	
C8	0.2944 (2)	0.45622 (14)	0.2665 (2)	0.0299 (5)	
C3	0.9512 (2)	0.03082 (14)	0.4782 (2)	0.0279 (5)	
C16	0.5904 (2)	0.27781 (15)	0.6063 (2)	0.0324 (6)	
C5	0.8725 (2)	0.16319 (15)	0.4734 (2)	0.0366 (6)	
H5	0.8856	0.2149	0.4992	0.044*	
C9	0.2624 (2)	0.38055 (16)	0.2287 (3)	0.0404 (7)	
H9	0.1813	0.3692	0.1888	0.048*	
C2	0.8399 (2)	0.01292 (15)	0.3977 (3)	0.0398 (7)	
H2	0.8257	−0.0379	0.3681	0.048*	
C11	0.4472 (2)	0.10091 (15)	0.3912 (2)	0.0321 (5)	
H11	0.4874	0.1128	0.4685	0.039*	
C10	0.3514 (2)	0.32131 (15)	0.2502 (3)	0.0395 (6)	
H10	0.3268	0.2708	0.2242	0.047*	
C4	0.9657 (2)	0.10903 (15)	0.5153 (2)	0.0376 (6)	
H4	1.0382	0.1248	0.5682	0.045*	
C1	0.7503 (2)	0.07089 (15)	0.3616 (3)	0.0373 (6)	
H1	0.6765	0.0568	0.3092	0.045*	
C6	0.5003 (3)	0.40610 (16)	0.3448 (3)	0.0447 (7)	
H6	0.5817	0.4157	0.3855	0.054*	
C17	0.8323 (3)	0.36663 (17)	0.3468 (3)	0.0437 (7)	
C14	0.3251 (2)	0.06625 (15)	0.1664 (2)	0.0319 (5)	
H14	0.2842	0.0567	0.0886	0.038*	
C7	0.4169 (3)	0.46839 (16)	0.3280 (3)	0.0469 (8)	
H7	0.4427	0.5180	0.3576	0.056*	
C19	0.7763 (4)	0.2828 (3)	0.0436 (4)	0.0746 (12)	
H19A	0.7051	0.2873	−0.0214	0.112*	
H19B	0.8476	0.2684	0.0148	0.112*	
H19C	0.7914	0.3324	0.0838	0.112*	
C18	0.7228 (5)	0.1284 (3)	0.0458 (5)	0.0992 (17)	
H18A	0.6943	0.0846	0.0842	0.149*	
H18B	0.7989	0.1146	0.0250	0.149*	
H18C	0.6611	0.1416	−0.0241	0.149*	
C12	0.3626 (2)	0.03931 (15)	0.3716 (2)	0.0344 (6)	
H12	0.3487	0.0102	0.4347	0.041*	
C13	0.2983 (2)	0.02117 (14)	0.2567 (2)	0.0298 (5)	

### Atomic displacement parameters (Å^2^)

**Table:**

	*U*^11^	*U*^22^	*U*^33^	*U*^12^	*U*^13^	*U*^23^
Co1	0.02248 (16)	0.02112 (16)	0.03429 (18)	0.00099 (12)	0.00529 (12)	0.00138 (13)
S3	0.0366 (4)	0.0522 (4)	0.0425 (4)	0.0033 (3)	0.0155 (3)	0.0038 (3)
S2	0.0528 (5)	0.0601 (5)	0.0505 (5)	−0.0048 (4)	0.0179 (4)	−0.0164 (4)
S1	0.0445 (5)	0.0713 (7)	0.1993 (16)	−0.0200 (5)	−0.0023 (7)	0.0688 (9)
N3	0.0229 (9)	0.0205 (10)	0.0362 (11)	0.0000 (7)	0.0053 (8)	0.0014 (8)
N2	0.0276 (10)	0.0266 (11)	0.0374 (12)	0.0033 (8)	0.0055 (9)	−0.0002 (9)
N1	0.0289 (10)	0.0263 (10)	0.0344 (11)	0.0059 (8)	0.0049 (8)	0.0017 (9)
O1	0.0297 (9)	0.0504 (12)	0.0399 (10)	−0.0013 (8)	0.0105 (8)	0.0048 (9)
N7	0.0391 (12)	0.0393 (13)	0.0380 (12)	0.0040 (10)	0.0054 (10)	0.0015 (10)
C15	0.0293 (12)	0.0246 (12)	0.0353 (13)	−0.0024 (9)	0.0075 (10)	0.0040 (10)
N6	0.0340 (12)	0.0319 (12)	0.0631 (16)	−0.0024 (10)	0.0083 (11)	−0.0004 (11)
C8	0.0277 (12)	0.0257 (12)	0.0364 (13)	0.0050 (9)	0.0079 (10)	0.0004 (10)
C3	0.0249 (11)	0.0260 (12)	0.0325 (12)	0.0051 (9)	0.0061 (9)	0.0009 (10)
C16	0.0292 (12)	0.0264 (13)	0.0394 (14)	−0.0001 (9)	0.0035 (10)	0.0015 (10)
C5	0.0342 (13)	0.0252 (12)	0.0450 (15)	0.0050 (10)	−0.0017 (11)	−0.0053 (11)
C9	0.0274 (13)	0.0291 (13)	0.0589 (18)	0.0036 (10)	−0.0012 (12)	−0.0063 (12)
C2	0.0325 (13)	0.0233 (12)	0.0560 (17)	0.0040 (10)	−0.0048 (12)	−0.0074 (12)
C11	0.0326 (13)	0.0286 (13)	0.0339 (13)	−0.0028 (10)	0.0053 (10)	0.0004 (10)
C10	0.0334 (13)	0.0227 (12)	0.0579 (18)	0.0029 (10)	0.0018 (12)	−0.0071 (12)
C4	0.0316 (13)	0.0296 (14)	0.0452 (15)	0.0038 (10)	−0.0037 (11)	−0.0054 (11)
C1	0.0263 (12)	0.0303 (13)	0.0491 (16)	0.0044 (10)	−0.0032 (11)	−0.0042 (12)
C6	0.0273 (13)	0.0306 (14)	0.069 (2)	0.0041 (10)	−0.0027 (13)	−0.0075 (13)
C17	0.0301 (13)	0.0312 (14)	0.0650 (19)	0.0001 (11)	0.0015 (13)	0.0108 (13)
C14	0.0309 (12)	0.0299 (13)	0.0326 (13)	−0.0054 (10)	0.0029 (10)	0.0001 (10)
C7	0.0348 (14)	0.0253 (13)	0.073 (2)	0.0028 (11)	−0.0029 (14)	−0.0119 (13)
C19	0.063 (2)	0.088 (3)	0.086 (3)	0.014 (2)	0.044 (2)	0.039 (2)
C18	0.111 (4)	0.095 (4)	0.109 (4)	−0.019 (3)	0.059 (3)	−0.054 (3)
C12	0.0374 (14)	0.0312 (13)	0.0352 (14)	−0.0073 (11)	0.0097 (11)	0.0056 (11)
C13	0.0259 (12)	0.0223 (12)	0.0411 (14)	−0.0025 (9)	0.0076 (10)	0.0002 (10)

### Geometric parameters (Å, º)

**Table:**

Co1—N7	2.080 (2)	C3—C3^ii^	1.506 (4)
Co1—N6	2.102 (2)	C5—C4	1.384 (3)
Co1—O1	2.1234 (19)	C5—H5	0.9300
Co1—N3	2.2187 (19)	C9—C10	1.390 (4)
Co1—N2	2.244 (2)	C9—H9	0.9300
Co1—N1	2.2551 (19)	C2—C1	1.392 (3)
S3—O1	1.5401 (19)	C2—H2	0.9300
S3—C19	1.765 (4)	C11—C12	1.388 (3)
S3—C18	1.766 (4)	C11—H11	0.9300
S2—C16	1.629 (3)	C10—H10	0.9300
S1—C17	1.623 (3)	C4—H4	0.9300
N3—C11	1.347 (3)	C1—H1	0.9300
N3—C15	1.348 (3)	C6—C7	1.388 (4)
N2—C10	1.336 (3)	C6—H6	0.9300
N2—C6	1.344 (3)	C14—C13	1.391 (4)
N1—C1	1.340 (3)	C14—H14	0.9300
N1—C5	1.343 (3)	C7—H7	0.9300
N7—C16	1.163 (4)	C19—H19A	0.9600
C15—C14	1.388 (3)	C19—H19B	0.9600
C15—H15	0.9300	C19—H19C	0.9600
N6—C17	1.152 (4)	C18—H18A	0.9600
C8—C9	1.379 (4)	C18—H18B	0.9600
C8—C7	1.393 (4)	C18—H18C	0.9600
C8—C13^i^	1.489 (3)	C12—C13	1.398 (4)
C3—C4	1.396 (3)	C12—H12	0.9300
C3—C2	1.396 (3)	C13—C8^iii^	1.489 (3)
			
N7—Co1—N6	93.76 (10)	C10—C9—H9	120.0
N7—Co1—O1	177.34 (8)	C1—C2—C3	120.2 (2)
N6—Co1—O1	87.22 (9)	C1—C2—H2	119.9
N7—Co1—N3	94.19 (9)	C3—C2—H2	119.9
N6—Co1—N3	171.85 (9)	N3—C11—C12	123.3 (2)
O1—Co1—N3	84.91 (7)	N3—C11—H11	118.4
N7—Co1—N2	85.58 (8)	C12—C11—H11	118.4
N6—Co1—N2	90.69 (8)	N2—C10—C9	123.9 (2)
O1—Co1—N2	91.94 (8)	N2—C10—H10	118.0
N3—Co1—N2	91.71 (7)	C9—C10—H10	118.0
N7—Co1—N1	90.49 (8)	C5—C4—C3	120.1 (2)
N6—Co1—N1	88.83 (8)	C5—C4—H4	119.9
O1—Co1—N1	92.00 (8)	C3—C4—H4	119.9
N3—Co1—N1	89.30 (7)	N1—C1—C2	123.8 (2)
N2—Co1—N1	176.00 (8)	N1—C1—H1	118.1
O1—S3—C19	105.89 (15)	C2—C1—H1	118.1
O1—S3—C18	105.07 (18)	N2—C6—C7	123.8 (2)
C19—S3—C18	99.4 (3)	N2—C6—H6	118.1
C11—N3—C15	116.7 (2)	C7—C6—H6	118.1
C11—N3—Co1	120.20 (16)	N6—C17—S1	179.5 (3)
C15—N3—Co1	123.09 (16)	C15—C14—C13	119.6 (2)
C10—N2—C6	115.9 (2)	C15—C14—H14	120.2
C10—N2—Co1	125.18 (17)	C13—C14—H14	120.2
C6—N2—Co1	118.89 (17)	C6—C7—C8	119.6 (2)
C1—N1—C5	115.8 (2)	C6—C7—H7	120.2
C1—N1—Co1	123.75 (16)	C8—C7—H7	120.2
C5—N1—Co1	120.37 (16)	S3—C19—H19A	109.5
S3—O1—Co1	115.11 (11)	S3—C19—H19B	109.5
C16—N7—Co1	161.0 (2)	H19A—C19—H19B	109.5
N3—C15—C14	123.6 (2)	S3—C19—H19C	109.5
N3—C15—H15	118.2	H19A—C19—H19C	109.5
C14—C15—H15	118.2	H19B—C19—H19C	109.5
C17—N6—Co1	166.3 (3)	S3—C18—H18A	109.5
C9—C8—C7	116.7 (2)	S3—C18—H18B	109.5
C9—C8—C13^i^	121.3 (2)	H18A—C18—H18B	109.5
C7—C8—C13^i^	122.0 (2)	S3—C18—H18C	109.5
C4—C3—C2	115.8 (2)	H18A—C18—H18C	109.5
C4—C3—C3^ii^	122.5 (3)	H18B—C18—H18C	109.5
C2—C3—C3^ii^	121.7 (3)	C11—C12—C13	119.8 (2)
N7—C16—S2	178.9 (3)	C11—C12—H12	120.1
N1—C5—C4	124.2 (2)	C13—C12—H12	120.1
N1—C5—H5	117.9	C14—C13—C12	117.1 (2)
C4—C5—H5	117.9	C14—C13—C8^iii^	122.2 (2)
C8—C9—C10	120.0 (2)	C12—C13—C8^iii^	120.8 (2)
C8—C9—H9	120.0		
			
N7—Co1—N3—C11	−21.36 (19)	N3—Co1—N7—C16	−124.4 (7)
N6—Co1—N3—C11	145.8 (5)	N2—Co1—N7—C16	−33.0 (7)
O1—Co1—N3—C11	161.15 (19)	N1—Co1—N7—C16	146.2 (7)
N2—Co1—N3—C11	−107.05 (18)	C11—N3—C15—C14	1.3 (4)
N1—Co1—N3—C11	69.08 (18)	Co1—N3—C15—C14	179.32 (19)
N7—Co1—N3—C15	160.68 (19)	N7—Co1—N6—C17	180.0 (10)
N6—Co1—N3—C15	−32.1 (7)	O1—Co1—N6—C17	−2.5 (10)
O1—Co1—N3—C15	−16.81 (19)	N3—Co1—N6—C17	12.8 (14)
N2—Co1—N3—C15	74.99 (19)	N2—Co1—N6—C17	−94.4 (10)
N1—Co1—N3—C15	−108.88 (19)	N1—Co1—N6—C17	89.5 (10)
N7—Co1—N2—C10	−107.9 (2)	C1—N1—C5—C4	−1.1 (4)
N6—Co1—N2—C10	158.4 (2)	Co1—N1—C5—C4	176.5 (2)
O1—Co1—N2—C10	71.1 (2)	C7—C8—C9—C10	−1.5 (4)
N3—Co1—N2—C10	−13.8 (2)	C13^i^—C8—C9—C10	179.1 (3)
N1—Co1—N2—C10	−118.5 (11)	C4—C3—C2—C1	−1.7 (4)
N7—Co1—N2—C6	69.0 (2)	C3^ii^—C3—C2—C1	178.4 (3)
N6—Co1—N2—C6	−24.8 (2)	C15—N3—C11—C12	0.4 (4)
O1—Co1—N2—C6	−112.0 (2)	Co1—N3—C11—C12	−177.7 (2)
N3—Co1—N2—C6	163.0 (2)	C6—N2—C10—C9	2.0 (4)
N1—Co1—N2—C6	58.4 (12)	Co1—N2—C10—C9	178.9 (2)
N7—Co1—N1—C1	116.1 (2)	C8—C9—C10—N2	−0.6 (5)
N6—Co1—N1—C1	−150.2 (2)	N1—C5—C4—C3	0.7 (5)
O1—Co1—N1—C1	−63.0 (2)	C2—C3—C4—C5	0.7 (4)
N3—Co1—N1—C1	21.9 (2)	C3^ii^—C3—C4—C5	−179.4 (3)
N2—Co1—N1—C1	126.6 (11)	C5—N1—C1—C2	0.1 (4)
N7—Co1—N1—C5	−61.4 (2)	Co1—N1—C1—C2	−177.5 (2)
N6—Co1—N1—C5	32.4 (2)	C3—C2—C1—N1	1.4 (5)
O1—Co1—N1—C5	119.5 (2)	C10—N2—C6—C7	−1.4 (5)
N3—Co1—N1—C5	−155.6 (2)	Co1—N2—C6—C7	−178.5 (3)
N2—Co1—N1—C5	−50.8 (12)	N3—C15—C14—C13	−1.8 (4)
C19—S3—O1—Co1	−127.1 (2)	N2—C6—C7—C8	−0.6 (5)
C18—S3—O1—Co1	128.3 (2)	C9—C8—C7—C6	2.0 (5)
N7—Co1—O1—S3	167.2 (18)	C13^i^—C8—C7—C6	−178.6 (3)
N6—Co1—O1—S3	55.48 (13)	N3—C11—C12—C13	−1.6 (4)
N3—Co1—O1—S3	−122.38 (13)	C15—C14—C13—C12	0.5 (4)
N2—Co1—O1—S3	146.08 (13)	C15—C14—C13—C8^iii^	−179.4 (2)
N1—Co1—O1—S3	−33.25 (13)	C11—C12—C13—C14	1.1 (4)
N6—Co1—N7—C16	57.4 (7)	C11—C12—C13—C8^iii^	−179.0 (2)
O1—Co1—N7—C16	−54 (2)		

Symmetry codes: (i) −*x*+1/2, *y*+1/2, −*z*+1/2; (ii) −*x*+2, −*y*, −*z*+1; (iii) −*x*+1/2, *y*−1/2, −*z*+1/2.

### Hydrogen-bond geometry (Å, º)

**Table:**

*D*—H···*A*	*D*—H	H···*A*	*D*···*A*	*D*—H···*A*
C1—H1···S1^iv^	0.93	2.82	3.596 (3)	141

Symmetry code: (iv) −*x*+3/2, *y*−1/2, −*z*+1/2.
